# Benefits of Virtual Reality Program and Motor Imagery Training on Balance and Fall Efficacy in Isolated Older Adults: A Randomized Controlled Trial

**DOI:** 10.3390/medicina58111545

**Published:** 2022-10-28

**Authors:** So-Hyun Kim, Sung-Hyoun Cho

**Affiliations:** 1 Department of Medical Sciences, Nambu University, 23, Cheomdanjungang–ro, Gwangsan–gu, Gwangju 62271, Korea; 2 Department of Physical Therapy, Nambu University, 23, Cheomdanjungang–ro, Gwangsan–gu, Gwangju 62271, Korea

**Keywords:** COVID-19, older adults, isolation, motor imagery training, virtual reality

## Abstract

*Background and Objectives*: for isolated older adults, alternative training indoors to maintain balance is essential; however, related studies are lacking. To improve the balance of isolated older adults and reduce their fear of falling, we aimed to examine the balance–keeping effect of a virtual reality (VR) program and motor imagery training (MIT) and propose training that could improve physical activity among older adults. *Methods*: a total of 34 older adults admitted to a convalescent hospital were assessed. VR (n = 12) and MIT (n = 10) groups completed their assigned intervention in six weeks, whereas the control group (CG) (n = 12) did not. The follow–up was performed after two weeks. *Results*: in group × time interactions, body center movement area, open and closed eyes balance scores, and fall efficacy were significantly different (*p* < 0.05). In contrast with the VR group, the MIT group did not show a significant difference in the open or closed eyes balance scores depending on the period. However, there was a significant difference between the MIT group and CG in the open eyes balance score post-test (d = 1.13, 95% confidence interval, 0.40–12.33). *Conclusions*: we propose VR and MIT as training methods to prevent physical weakness in isolated older adults.

## 1. Introduction

Due to the severe coronavirus (SARS-Cov-2) infection, older people are considered vulnerable [1]. Currently, older adults are engaging in protective measures, such as self-isolation. However, quarantine measures act as risk factors for the possibility of falling despite the benefits [2]. Even in healthy older people, physical weakness due to the natural aging process is a factor that cannot be ignored [3,4]. Furthermore, emotional anxiety in older adults increases due to social isolation and causes a decline in their physical activity [5].

Decreased physical activity can cause falls among older adults, exposing them to the risk of death or serious injuries from falls [6]. Therefore, maintaining balance in older adults is essential, and promoting and inducing physical activity is paramount [7]. Training during COVID-19 isolation ensures improved levels of physical activity and can play an important role in mental and physical health [8].

Among older adults, balance and body sway measures are strong predictors of fall risk [9,10]. In the measurement of body sway, the area of the center of pressure (COP) is most consistently associated with falls [11]. The classification of older adults with a high risk of falling was verified by recording static equilibrium [12]. Older people find it more difficult to maintain a static posture than walking [13] and show more body sway than healthy adults during tasks that require balance [14]. Therefore, static balance management acts as a factor that can lower the risk of falls in older adults. In addition, reducing the fear of falling can provide benefits that accompany safe movements and increase activities of daily living [15].

Regular exercise can strengthen the immune system of older adults and offer various health benefits, such as lowering the risk of chronic diseases [16]. Therefore, the World Health Organization (WHO) recommends that older adults learn simple physical exercises that can be performed indoors to reduce boredom and maintain mobility during quarantine [17]. Furthermore, for indoor physical activity, it is important to reduce costs and ensure safety [18].

Virtual reality (VR) programs [19] and motor imagery training (MIT) [20] can be performed indoors to ensure safety. VR programs can help express real-world behavior by providing direct visual and auditory feedback in three-dimensional virtual space [21]. The Nintendo Wii balance board (WBB), a VR program, provides safe, adaptable, and low-cost balance training for older adults [22]. The WBB demonstrates reliability (*r* = 0.75–0.91) for the COP associated with static balance [23] and shows that VR is effective in improving balance in older adults [24,25]. Moreover, as an alternative to reducing social isolation, a VR program that combines exercise and games could attract the interest of older adults [26].

MIT refers to internally perceiving motor imagery (MI) that imagines motions without physical movements and simulates them mentally [27]. Just by performing MIT, a reaction similar to that of the active area of the brain that activates during physical training can be obtained, resulting in similar benefits to physical training [28]. Furthermore, the greatest advantages of MIT are that it is not costly and improves performance by providing individuals with arousal and concentration strengthening [29]. Regardless of age, older adults are as effective as young adults in forming MI [30]. Therefore, MIT can be an alternative to improve the balance ability of older adults and prevent a decrease in physical activity [31].

However, experimental studies to improve the balance ability of isolated older adults are insufficient. Therefore, this study aimed to evaluate whether the continuation of improving the physical function of isolated older adults and the positive effects of training by conducting an additional follow-up survey differed from previous studies. Furthermore, to improve the balance of isolated older adults and reduce the fear of falling, this study examined the effects of a VR program and MIT and proposed an alternative training for physical activity in older adults. This study mainly aimed to determine the effects of VR and MIT on static balance ability (body center movement area and open and closed eyes balance sensory scores) and fall efficacy among isolated older adults.

The hypotheses of this study are as follows: (1) VR and MIT will be effective in improving the static balance ability of isolated older adults, and (2) VR and MIT will be effective in improving fall efficacy among isolated older adults.

## 2. Materials and Methods

### 2.1. Design and Participants

This study was conducted for eight weeks and included 36 older adults aged 65 years and older who were admitted to a convalescent hospital in Gwangju, Korea. The estimated sample size was calculated using G*Power 3.19 [32], with a significance level of 0.05, effect size (*d*) of 0.8, and power (1 − *β*) assumed to be 0.95 [33,34]. Considering a dropout rate of 20%, we assigned 12 people to each group.

After receiving approval from the convalescent hospital director to recruit participants, a recruitment notice was posted in the ward, and the application for recruitment was conducted for four weeks. Among the hospitalized patients who applied, the attending physician confirmed those for whom there was no risk in participation, and then the participants were selected.

The inclusion criteria were as follows: (1) intact vestibular function and vision and no hearing impairments; (2) the capability of daily life and independent walking; (3) no musculoskeletal lesions and history of surgery within the past six months; (4) No performance of regular exercise over the past six months that could affect the experiment; (5) isolation for at least three months; and (6) voluntary agreement to participate in this study.

The exclusion criteria were as follows: (1) Korean mini-mental status examination (MMSE–K) score of 24 or less, (2) Vividness of movement imagery questionnaire (VMIQ) score of 2.26 or more, (3) use of drugs that can affect balance ability, (4) performance of regular exercise over the past six months that could affect the experiment, (5) progressive or neurological disorders (stroke, dementia, Parkinson’s disease, and others), and (6) No wish to participate in this study.

Participants who voluntarily agreed to participate in the study were given explanations and guidance. The purpose of the study and the inconvenience that could occur by participating in the study, the confidentiality of identity and protection of privacy, the possibility of discontinuing the study if desired, and the absence of disadvantages during the study period were explained, and written consent was acquired before participation in the study.

The intervention model was factorial, and the participants were randomly assigned to one of three groups following simple randomization procedures (computerized random numbers). The researcher enrolling and assessing participants was blinded to the allocation sequence in sequentially numbered, opaque, sealed, and stapled envelopes. Corresponding envelopes were only opened after the enrolled participants completed all baseline assessments to allocate the intervention.

A single-blind study was conducted to reduce bias during the study period. The lead researcher could not be blinded to the group allocation of participants during test administration but was blinded to the group allocation during the data analysis process. In addition, the evaluation was performed by a blinded assistant researcher unaware of the group allocation. Other researchers not involved in the study prepared and deidentified the data using computer-generated random number sequencing before data were analyzed by the lead researcher.

**Figure 1:**
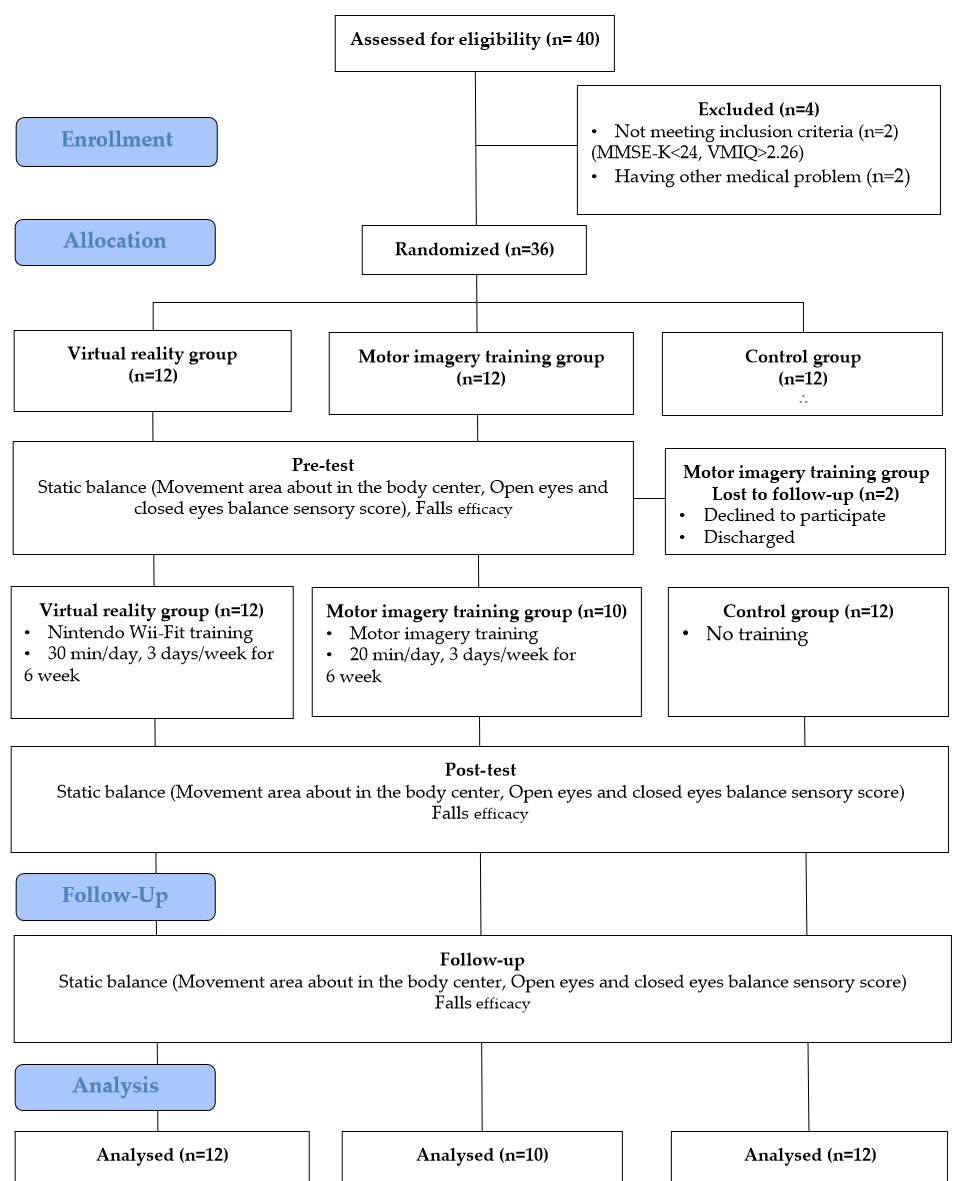
Research flow diagram.

There were two dropouts; therefore, the study was conducted with 34 participants (Figure 1). The primary outcome was the body center movement area, while the secondary outcomes were open and closed eyes balance scores and fall efficacy. Intervention programs assigned to each group were implemented for six weeks. Additional follow-up was performed two weeks after the completion of training.

The follow-up was conducted as follows: all participant groups maintained their daily life standards for two weeks after the end of training. Any intervention or independent exercise was controlled. The absence of intervention or exercise was confirmed through conversations with participants in the morning and afternoon. In addition, a caregiver residing in the inpatient ward 24 h a day was asked to monitor the participants.

This study was approved by the Institutional Review Board of Nambu University (IRB:1041478-2017-HR-016) and conducted in accordance with the ethical standards of the Declaration of Helsinki. All participants understood the study’s aim and provided written informed consent. This study included a clinical trial registration number (KCT0006053).

### 2.2. Patient Safety Precautions and Compliance

The chief researcher subscribed to liability insurance and conducted the experiment in a location with a surveillance camera. It was announced that in the event of unpredictable physical or mental risks, the researcher would take legal responsibility and compensate participants for any damage. During the study, a space for first aid was secured, and the study was conducted during the hospital’s working hours to ensure that if an emergency occurred, the medical staff could arrive at the emergency site within 1 min.

Soft flooring was provided to prevent falls, and cleaning tools were provided in case of liquid spills. Patients were requested to wear sneakers, and chairs were arranged in case participants needed rest during treatment. Two assistant researchers who did not participate in the experiment were present during training or evaluation to prevent falls. Hand sanitizers and masks were always provided to prevent the risk of COVID-19 infection, and the researcher experimented while wearing face shields, KF-94 masks, gloves, and protective clothing. Each participant was scheduled individually to avoid coming into contact with one another.

During the study, it was clearly explained in advance that participants could withdraw from the study at any time in consultation with the researcher if they chose to. In addition, the researcher encoded all data, assigned a password, and stored the data on a separate USB drive to protect participants’ personal information. Furthermore, the study participants received a full explanation of the risks and benefits of the experiment, and a token of appreciation was provided after the intervention and evaluation.

To ensure compliance with the study procedures, the lead researcher had the assisting researchers participate in the experiment after sufficiently educating them about the procedure and regulations. All experimental procedures were conducted under the lead researcher’s guidance to ensure regulations compliance further.

### 2.3. Intervention Method

**Figure 2:**
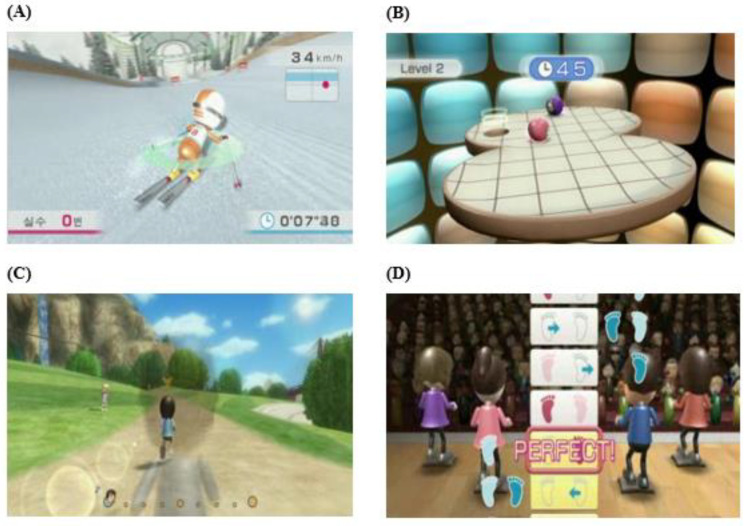
Virtual reality exercise program. (**A**) Balance ski; (**B**) Table tile; (**C**) Jogging; (**D**) Rhythm step.

The VR program was implemented using a Wii Fit game program, Wii-only software released by Nintendo Inc., Kyoto, Japan. It was performed using a Wii balance board (WBB) and Wii remote control. For the program contents, “Balance ski” and “Table tile” were implemented to improve participants’ weight transfer, weight support, and speed. In addition, to improve aerobic capacity, muscular endurance, and muscle strength, we chose “Jogging” and “Rhythm step,” which are similar to climbing stairs (Figure 2). The VR program was conducted for six weeks, three times a week, for 30 min a day, with 5 min of warm-up stretching, 5 min of each program, and 5 min of rest.

MIT was conducted at 6 p.m. in a quiet location with limited access to outsiders to promote participants’ concentration. The light from outside was blocked, and minimal lighting was used to avoid visual confusion for 20 min. Participants sat comfortably on a chair with armrests. Previous evidence indicates that auditory cues are the most effective for older adults; therefore, participants were requested to close their eyes and imagine it through the therapist’s verbal instructions [35]. Contents of MIT were modified and applied accordingly for participants with stroke to suit their specific needs [36]. MIT can be divided into the motor- and visual-sensory imagery training. In the motor-sensory imagery training, the participants were asked to feel the pressure on the soles of their feet, weight shifting, and a sense of position during weight bearing. Visual-sensory imagery training allowed the participants to imagine observing their body movements from the perspective of a third person. The participants were instructed to raise their hands if they had questions in the middle of the training to avoid disturbing concentration. MIT was conducted for 20 min a day, three times a week, for six weeks. Since extended training sessions negatively affect participants, the recommended training time was maintained within 20 min [37]. A relaxation state of 150 s was induced before and after the start of MIT. A total of 20 min of main training was performed using motor- and visual-sensory imagery training for 15 min. The control group (CG) did not undergo any treatment intervention.

### 2.4. Measurement

The Gaitview AFA-50 system (alFOOTs, Seoul, Korea), which measures body center movement area with 2304 pressure sensors on the foot scan board, was used to obtain the open and closed eyes balance scores. The reliability of this system has been previously demonstrated [38]. The static balance measurement method was as follows: Before the measurement, the motion was briefly practiced for 30 s, and the participant stood upright as much as possible on the measuring device while barefoot, gazing at a location marked in advance. Next, the body center movement area was measured by maintaining a static posture for 10 s. The balance score for the open and closed eyes (eyes were closed for 3 s before measurement) was also measured for 20 s in the same way.

Fear of falling was measured using the Tinetti Falls Efficacy Scale (*r* = 0.71) [39]. A total of 10 questions measured each participant’s confidence level that they would not fall when performing daily activities. In the daily movements of each item, “very confident” was assigned one point, while “not confident” was assigned 10 points. The measurement scores ranged from the lowest (10 points) to the highest (100 points). The lower the score, the higher the fall efficacy. A score of 70 or higher was considered to indicate a high risk of falling.

### 2.5. Statistical Analysis

Data analysis of the research results of this study was performed using IBM SPSS version 26.0 for Windows (IBM Corp., Armonk, NY, USA). The mean, standard deviation, and descriptive statistics for each measured variable were calculated. The participants’ general characteristics were analyzed using a one-way analysis of variance (ANOVA). Statistical processing was performed using a two-way repeated measures ANOVA to compare the changes according to the period between the pre-, post-, and follow-up tests between each group. The main effect of the group was evaluated using one-way ANOVA, and the main effect of time using one-way repeated measures ANOVA. Post-tests for statistically significant differences were performed using Scheffe’s method. The statistical significance level (α) was set at *p* < 0.05.

## 3. Results

### 3.1. Characteristics of Participants

A total of 36 participants were included in this study; however, one in the MIT group refused to participate, and another was discharged from the hospital. Therefore, 34 people participated in the final experiment. There were no significant differences in the homogeneity and normality tests between the groups (*p* > 0.05) (Table 1).

### 3.2. Comparison of Static Balance Ability of Each Group

Body center movement area (*p* = 0.010), open eyes (*p* = 0.000), and closed eyes (*p* = 0.007) balance sensory scores all had statistically significant differences in the interaction effect of group × time. The body center movement area was significantly different in the main effect test during the study period (*p* = 0.002). In addition, there was a significant difference in the closed-eyes balance sensory score in the interaction according to the period (*p* = 0.007) (Table 2).

**Figure 3:**
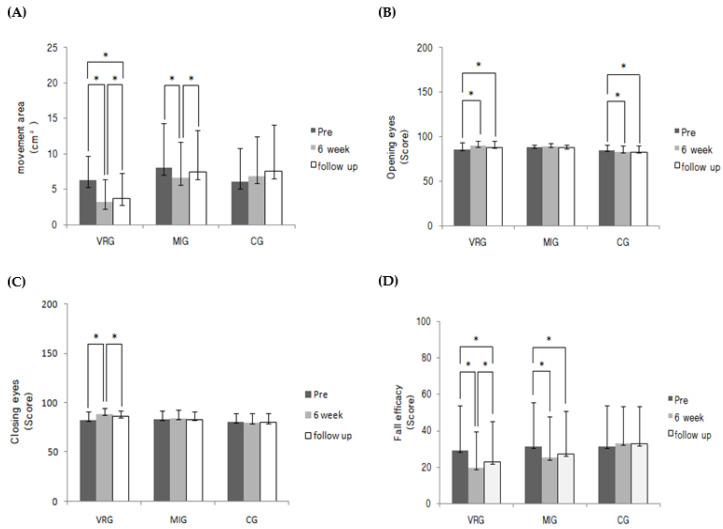
Comparison of each group according to the period. (**A**) Body center movement area; (**B**) Balance sensory with eyes kept open; (**C**) Balance sensory with eyes kept closed; (**D**) Falls efficacy score. * *p* < 0.05.

In contrast with VR programs, which showed significant differences in all periods, MIT did not show significant differences in the open- or closed-eyes balance sensory scores (*p* > 0.05). However, in the open-eyes balance sensory score, the VR (*p* = 0.02, d = 1.08, mean difference [MD] = 6.75, 95% confidence interval [CI]: 1.06–12.44) and MIT (*p* = 0.04, d = 1.13, MD = 6.37, 95% CI: 0.40–12.33) groups showed significant differences in the post-test (*p* < 0.05) (Table 2). Furthermore, the CG showed significant negative changes in the pre- and post-test (d = −0.32, 95% CI: 0.3–3.7) and the pre-test and follow-up (d = −0.34, 95% CI: 0.5–3.7) (Figure 3). Therefore, although there was no significant difference in MIT, it was confirmed that it positively affected static balance compared with CG.

### 3.3. Comparison of Changes in Fall Efficacy

There was a statistically significant difference in the group × time interaction (*p* = 0.000). There was also a significant difference in the main effect test according to the experimental period (*p* = 0.000) (Table 2). In the VR program, there was a significant decrease in fear of falling at all periods (*p* < 0.05), and post-follow-up (d = −0.09, 95% CI, −4.4–0.2) was excluded for MIT. In addition, there was a significant decrease at all time points (*p* < 0.05) (Figure 3).

## 4. Discussion

Older adults in convalescent hospitals face complete isolation due to the prolonged COVID-19 lockdown. In convalescent hospitals, it is strongly recommended that older patients be protected from outside exposure to infectious diseases [18]. However, during quarantine periods, even physical well-being may be weakened owing to lockdowns, curfews, and the psychological anxiety of being socially isolated [5,18]. Older adults may be at risk of falling due to reduced physical activity, which is a disadvantage of isolation [5,6]. Previous evidence indicates that physical rehabilitation interventions are associated with significant improvements in physical and mental function without increasing the risk of death in isolated older adults [40,41]. Therefore, in this study, a VR program and MIT were conducted considering their cost efficiency and sustainability of physical function improvement to preserve the level of physical activity in isolated older adults.

This study’s results confirmed that the VR program was effective for all static balance abilities and fall efficacy. However, MIT did not significantly differ between open and closed eyes balance conditions. These results should be interpreted considering that older adults tend to use an ankle joint strategy rather than a hip joint strategy to maintain balance [42]. In a muscle strength study that used a VR program, it was found that the ankle muscles were strengthened more effectively than the hip muscles [43]. This finding and our current results are consistent with those of previous studies that suggested the utility of WBB, which is mainly used as a reference for static balance data in older adults [44]. A VR-related study similar to this study reported that the decrease in the moving area of the body influenced the balance ability of older adults [45], thus reducing the fear of falls with an average improvement of 11% in fall efficacy [22]. Moreover, previous studies aimed to improve postural control with visual feedback in older adults demonstrated that VR programs are potentially associated with visual feedback [46]. The VR program was thought to be more effective than MIT during short-term training due to visual feedback’s influence. Consistent with our findings, a previous study that used a VR program in a nursing home reported improved static balance ability under both open and closed eyes conditions [47].

Conversely, previous studies of MIT differed from our study in that a significant difference was observed in the balance ability of older adults between open and closed eyes conditions [48,49]. This difference may be due to variations in the evaluation of scores, as the static balance ability evaluation used in the previous studies was limited to one-leg standing. In this study, despite the same static balance ability measurement, there was a significant difference in the body center movement area but not in the open- or closed-eyes balance scores. Evaluation tools, such as COP distance and Timed Up and Go (TUG), are typically used to evaluate the static and dynamic balance ability improvements among older adults over time with MIT [34,50]. This is consistent with our results; however, according to the aforementioned differences in results, it is thought that a multi-faceted evaluation approach is needed to evaluate the balance ability of older adults in future studies.

Notably, no significant difference was found between the open and closed eyes conditions after MIT; however, better results were obtained in the mean difference pre- and post-intervention. Therefore, longer interventions may be required to improve accurate open and closed eyes balance scores.

Apparently supporting these effects, we observed that repetitive MIT differed significantly in the open eyes balance score in the CG. Moreover, the score in the CG without any intervention decreased negatively with time. The results of a study of MIT with older adults were consistent with ours in comparing balance and fall efficacy with CG [50]. In contrast, another study that measured TUG in a single session, although not a static balance study, found no differences between the MIT group and CG [34]. Comparing static and dynamic balance can be difficult; however, the conflicting results may be due to differences in intervention duration. Furthermore, most of the previous studies that included older adults reported only significant improvement in static balance ability compared with controls [20], thus limiting direct comparison with our study’s results. Alternatively, regarding the visual dependence of older adults, the inactivation of subcortical areas responsible for balance ability during MI without visual input was observed [51]. These results indicate that there may be no difference between the MIT group and CG in our study. However, in contrast with this study, MI was performed in previous studies only by imagination without auditory stimulation and based on the first-person point of view. Moreover, during MI, the subcortical area showed inactivation; however, the premotor and prefrontal cortices, which are responsible for posture control and executive functions, showed activation. Therefore, MI is thought to have a more positive effect on static balance ability than no intervention. In addition, in a study that compared the static balance in the open and closed eyes conditions in young and older men, older men had increased difficulty in static balance with closed eyes than with open eyes [52]. Therefore, similar to our findings, it is thought that it may be more difficult to maintain and improve static balance with eyes closed during interventions, as older adults rely more on visual input to ensure balance. In addition, the visual contribution to postural stabilization is significantly greater in non-fall-experienced individuals than in fall-experienced individuals [53]. This suggests that, despite the CG undergoing an open-eyes sensory assessment, a decreased score may lead to a higher risk of subsequent falls if adequate interventions are not administered. Therefore, MIT can be an attractive alternative to physical methods for isolated older people using VR programs.

Notably, MIT reduced the area of body sway and the fear of falls among older participants [50,54], and a meta-analysis study proved that MIT is an effective alternative for improving balance ability in older adults [20]. MI is associated with activating brain areas involved in gait regulation and fear responses [55]. Therefore, older people afraid of falls are predicted to have a lower MI and a higher risk of falls. Balance and fall efficacy are closely related [56], and our results indicate that repetitive MIT improves balance in older adults. Therefore, balance and fear of falling should be considered together in older adults. In addition, a VR program and MIT, both of which can continue to improve physical function in older adults, should be provided to isolated older adults.

This study had some limitations. First, the small sample size makes it difficult to generalize the results. Second, older adults isolated at home were not considered. Third, because the direct fall incidence rate and prevention associated with training were not presented, they should be addressed in future studies. Fourth, in addition to the small sample size, the outcomes of this study, which were based on only the pre- and post-test results obtained after the intervention, did not predict the follow-up duration of the effect.

## 5. Conclusions

Our results indicate that the VR program and MIT improve balance and fear of falling in isolated older adults. Future research to ensure the physical level of socially isolated older people must approach various training methods to improve physical activity considering cost-effectiveness and environmental conditions. This study proposes the VR program and MIT as effective interventions for improving physical function in isolated older people and preventing falls.

## Figures and Tables

**Table 1:** General characteristics of the participants (n = 34).

Groups (n)	Age (Years)	Height (cm)	Weight (kg)	BMI (kg/m^2^)	MMSE–K	VMIQ	Sex (M/F)
VRG (12)	75.75 ± 10.15	157.17 ± 9.48	58.67 ± 12.15	23.82 ± 5.23	26.17 ± 1.53	1.60 ± 0.33	5/7
MIG (10)	83.10 ± 5.24	156.20 ± 10.49	54.70 ± 9.15	21.88 ± 2.73	25.60 ± 1.90	1.89 ± 0.31	4/6
CG (12)	80.75 ± 6.03	157.33 ± 10.53	59.75 ± 9.98	24.90 ± 4.30	25.83 ± 1.59	1.76 ± 0.34	6/6
F	2.753	0.039	0.672	1.366	0.325	2.207	
p	0.079	0.962	0.518	.270	0.725	0.127	

mean ± SD, mean ± standard deviation; VRG, virtual reality group; MIG, motor imagery group; CG, control group; BMI, body mass index; MMSE–K, Korean Mini-Mental State Examination; VMIQ, Vividness of Movement Imagery Questionnaire; M/F, Male/Female.

**Table 2:** A comparison of balance and falls efficacy scores during the intervention in each group (body center movement area unit: cm^2^, balance sensory related to people eyes and falls efficacy unit: score).

Variables	Group	Pre-Test (Week 0)	Post-Test (Week 6)	Follow-Up (Week 8)	Mean Difference (95% CI) Effect Size (*d*)			F (*p*)		
		M ± SD	M ± SD	M ± SD	Pre-Test Compared with Post-Test	Pre-Test Compared with Follow-Up	Post-Test Compared with Follow-Up	Group	Time	Group × Time
Body center movement area	VRG	6.3 ± 3.3	3.26 ± 3.13	3.76 ± 3.54	3.1 (1.0 to 5.1) 0.95	2.6 (0.5 to 4.6) 0.75	−0.5 (−1.0 to −0.0) −0.15	1.205 (0.313)	7.822 (0.002 *)	3.627 (0.010 *)
	MIG	8.0 ± 6.3	6.63 ± 5.05	7.44 ± 5.85	1.4 (0.0 to 2.8) 0.25	0.6 (−0.6 to 1.8) 0.10	−0.8 (−1.5 to −0.1) −0.15			
	CG	6.1 ± 4.7	6.87 ± 5.54	7.54 ± 6.51	−0.8 (−1.6 to 0.1) −0.15	−1.4 (−3.6 to 0.7) −0.25	−0.7 (−2.2 to 0.9) −0.11			
Balance sensory with eyes kept open	VRG	85.9 ± 7.9	89.6 ± 5.8 ^†^	88.1 ± 7.3	−3.7 (−6.5 to −0.8) 0.53	−2.2 (−3.6 to −0.8) 0.28	1.5 (−0.5 to 3.5) −0.23	2.483 (0.100)	2.848 (0.066)	6.122 (0.000 *)
	MIG	88.1 ± 2.5	89.2 ± 3.8 ^†^	87.3 ± 3.6	−1.1 (−2.8 to 0.6) 0.34	0.8 (−1.9 to 3.5) −0.26	1.9 (−0.4 to 4.2) −0.51			
	CG	84.8 ± 5.6	82.8 ± 6.8	82.8 ± 6.7	2.0 (0.3 to 3.7) −0.32	2.1 (0.5 to 3.7) −0.34	0.1 (−0.7 to 0.9) −0.01			
Balance sensory with eyes kept closed	VRG	82.7 ± 8.2	88.2 ± 6.6	86.3 ± 5.7	−5.5 (−9.6 to −1.4) 0.74	−3.6 (−7.7 to 0.5) 0.51	1.9 (0.9 to 2.9) 0.31	1.344 (0.276)	5.860 (0.007 *)	3.888 (0.007 *)
	MIG	83.3 ± 9.0	84.3 ± 8.8	83.1 ± 8.4	−1.0 (−2.7 to 0.7) 0.11	0.2 (−0.6 to 1.0) −0.02	1.2 (−0.6 to 3.0) −0.14			
	CG	80.8 ± 8.9	80.0 ± 9.6	80.2 ± 9.2	0.8 (−0.2 to 1.9) −0.09	0.7 (−0.3 to 1.7) −0.07	−0.2 (−1.1 to 0.8) 0.02			
Falls efficacy	VRG	29.1 ± 24.8	19.7 ± 19.9	22.3 ± 22.0	9.4 (5.4 to 13.5) 0.42	6.2 (3.7 to 8.7) 0.26	−3.3 (−5.1 to −1.4) −0.15	0.431 (0.653)	15.724 (0.000 *)	7.509 (0.000 *)
	MIG	31.2 ± 24.3	25.1 ± 22.4	27.2 ± 23.6	6.1 (3.9 to 8.3) 0.26	4.0 (2.0 to 6.0) 0.17	−2.1 (−4.4 to 0.2) −0.09			
	CG	31.2 ± 22.5	33.0 ± 20.2	32.7 ± 20.6	−1.8 (−4.2 to 0.5) −0.09	−1.5 (−3.3 to 0.3) −0.07	0.3 (−0.9 to 1.6) 0.02			

* *p* < 0.05; ^†^ Significant differences between intervention groups compared with the control group (*p* < 0.05); mean ± SD—mean ± standard deviation; 95% CI, 95% confidence interval; VRG, virtual reality group; MIG, motor imagery group; CG, control group.

## Data Availability

All data relevant to the study are included in the article.
